# Recent ASA Presidents and ‘Top’ Journals: Observed Publication Patterns, Alleged Cartels and Varying Careers

**DOI:** 10.1007/s12108-016-9330-0

**Published:** 2016-09-14

**Authors:** Jennifer Platt

**Affiliations:** Freeman Building, University of Sussex, Brighton BN1 9QE, Sussex, UK

**Keywords:** American Sociological Association presidents, *American Sociological Review*, *American Journal of Sociology*, Top journal, Cartel

## Abstract

It has been common for studies presented as about American sociology as a whole to rely on data compiled from leading journals (*American Sociological Review* [ASR] and *American Journal of Sociology* [AJS]), or about presidents of the American Sociological Association [ASA], to represent it. Clearly those are important, but neither can be regarded as providing a representative sample of American sociology. Recently, Stephen Turner has suggested that dominance in the ASA rests with a ‘cartel’ initially formed in graduate school, and that it favors work in a style associated with the leading journals. The adequacy of these ideas is examined in the light of available data on the last 20 years, which show that very few of the presidents were in the same graduate schools at the same time. All presidents have had distinguished academic records, but it is shown that their publication strategies have varied considerably. Some have had no ASR publications except their presidential addresses, while books and large numbers of other journals not normally mentioned in this context have figured in their contributions, as well as being more prominent in citations. It seems clear that articles in the leading journals have not been as closely tied to prestigious careers as has sometimes been suggested, and that if there is a cartel it has not included all the presidents.

## The Presidents and the Journals

Historians and sociologists of sociology have often characterized whole national sociologies, especially in the US, without giving much attention to the methodological issues involved. Empirical data used for this purpose, as when the extent to which qualitative or quantitative methods are used is measured, have commonly been on articles in the recognized major journals; sometimes American Sociological Association [ASA] presidents and their presidential addresses have been used in the same way. It is generally taken as self-evident that the discipline and its publications are stratified, and that the *American Sociological Review* [ASR] holds the top position among journals, closely followed by the *American Journal of Sociology* [AJS]. Stephen Turner (2014) has recently argued that there is a distinctive ASR/AJS style of sociology associated with an elite also dominant in the ASA, and that the leading journals play an increasingly key role in the social system of the discipline. Here we focus on what can be said about presidents of the ASA, and/or leading journals, as potentially representative of US sociology in general, or as dominated by such a clique, and the authorship of ASR/AJS articles as a crucial currency in the pursuit of power in the ASA.

It is obvious, if seldom explicitly noted, that neither articles in leading journals nor presidents are representative samples of the larger whole of US academic sociology’s publications and/or members. Few people can publish in the limited space of what are commonly recognized as the leading journals, and even counting the whole membership of the ASA leaves out sociologists who have not joined.^1^ Its presidents are, however, representative of the membership in the sense that they, like other ASA officers, are chosen by election.^2^ The logic of much previous work’s approach differs from conventional sampling, resting on assumptions about the normative status of associational office or leading journals. Some quotations to exemplify such approaches to journals:

- ‘Given both the high ‘intensity’ and ‘extensity’ of each journal’s prestige, we hoped that an accurate portrayal of the major theoretical and methodological orientations existent within sociology would be forthcoming.’ (Snizek 1975: 418)
- ASR is ‘the most prestigious journal within the discipline’, so ‘should^3^ reflect the highest and most rigorous peer review process’ (Wells and Picou 1981: 80)
- They are commonly used and peer reviewed, so give ‘a picture of what could be considered to be research endorsed by the discipline’ (MacInnes et al. 2012)

Similar issues are raised by some treatment of presidential addresses as representative, as exemplified in these further quotations:

‘… reading of the addresses offers a first-hand access to the minds of those whom the members of the Association chose to represent the sociological body politic to the other disciplines, to the society at large and, first and foremost, to the membership of the Association.’ (Kubat 1971: 2)

‘... by examining the views of … ASA presidents as indicated in their annual addresses… general conclusions are drawn regarding the functions of professional sociology as a whole.’ (Kinloch 1981: 2-3)

It is obvious that the justification by high prestige conflicts directly with that which rests on claims to typicality. When, for example, only ASR data are used, this ignores the fact that most US sociologists have never published a paper there - and there has been much criticism from the constituency of its perceived intellectual character.^4^ Whether or not those most successful and dominant within the system are seen as a cartel excluding non-members, it tends to be assumed that there are interdependent parts which interlock to make the whole system: articles written by the elite are the kinds of article that get published in ASR/AJS, to have articles in ASR/AJS wins a place within the occupational system which cannot be won without that, holders of those places write more of that kind of article, and staff the refereeing system….

There are well-known patterns of stratification in the American discipline among both individuals and departments or universities,^5^ and data on the elite - treated as such, not as representatives of a wider constituency - are both of interest in themselves and a necessary part of the complete picture. It can indeed be taken for granted that the presidency of the ASA is a high distinction, but that does not make its holders representative in the sense of typicality. Turner (2014) brings a rather different perspective to bear, where the alternative to representativeness is not elite superiority, but what he refers to as a disciplinary cartel; he argues that the social system of US sociology has become one in which the journal system, with its heavy stress on the ASR and AJS, and with the less common inclusion of *Social Forces* (SF), plays a key role:

‘The leadership of the ASA is not “representative” of American sociology. It consists of a group of friends, usually connected with one another for decades, normally since graduate school, and is exclusive. Its insiders allocate positions, responsibilities and power to one another…’ (Turner 2014: 65)^6^

‘It might seem that the … prestige hierarchy and the focus on the ASR/AJS system… should not survive… this is a system that no longer depends on convictions or common purpose, or on the idea of a program of advancing sociology as a science. It rests on the mechanical foundation of the review system.’ (Turner 2014: 119)

The focus in this paper is on the presidents as such, and available data to explore the adequacy of various pictures of the system are used, with special attention to relations between the journals and presidents.

It is interesting that studies which discuss such issues have seldom felt it necessary to provide data to show that ASR and AJS *are* intellectually the leading journals^7^; we all ‘know’ that already. How could we measure the achievement of that status? There are customary, if not always good, criteria which can be used for evaluating departments and universities, but these often introduce an element of circularity by relying on measurements of reputation; for journals this commonly takes the form of prestige, rather than the *grounds* for the prestige. An obvious possibility would be the merits of the articles they publish, assessed individually in the usual way (whatever that is).^8^ It is not very convincing to suggest that the peer-refereeing system is sufficient to ensure that the articles selected by ASR and AJS are of the highest standards, because they are by no means the only journals which have such a system. Moreover it has been found that, when individual articles were categorized, ‘highly regarded articles appear in less highly regarded journals… while less highly regarded articles appear in highly ranked journals’ Teevan (1980:112).^9^ Here no assumption is made about the journals’ ‘real’ superiority, but their perceived superiority is taken as given.

Some basic descriptive material on the twenty most recent presidents is presented, and we go on to consider what this can show for such interpretations.

## Methods

Two samples are used:

- The complete set of the ASA presidents from 1995 to 2014. As far as possible, full cvs with publication details have been obtained for each president, and those are supplemented by other sources such as issues of the ASA’s *Guide to Graduate Departments*, election statements, and a few autobiographical publications.
- Something nearer to a representative sample of the discipline is drawn from the ASA’s *Cumulative Index of Sociology Journals 1971–1985* (Lantz 1987); this indexes all ASA journals, plus AJS and SF, offering material which can be treated as in some sense a sample of authors, of articles and of journals.^10^ It is unfortunate that the period covered by it ends early in the careers of some members of the presidential group, but it can reasonably be seen as covering some of their formative years. These Index data are nearer to a set of the general sociological public’s articles than any other sources identified – which does not make them very near - and so are occasionally used for that below.^11^

From here on ASR, AJS and SF journals and articles are referred to as ‘top’ (without repeated quotation marks), and all other papers become ‘non-top’. Not dealt with separately is what might be seen as the journal middle classes, of longstanding and well-respected but not ‘top’ journals; this includes several of the US regional ones, the *British Journal of Sociology*, and the British Sociological Association’s *Sociology*, with general remits, and some very well established more specialist journals such as *Social Problems* and the ASA’s *Sociology of Education*, *Social Psychology Quarterly*, and *Journal of Health and Social Behavior*. This is where Turner in effect places SF, since he treats only ASR and AJS as the top journals; SF is included as top by this paper since it has been included by so many earlier writers. (Perhaps there is a historical change here?) However, including those, perhaps in a category between top and non-top as used in this paper, would still leave out a large number of other journals - more specialized within sociology, on new topic areas, or less specifically sociological.

Some basic facts about the presidents studied, also drawn on below, are given in Table 1.

**Table 1:** 20 years of ASA Presidents^a^

President	Born	PhDdate	Presidency date	Institutional affiliation when became President^b^
Etzioni, Amitai	1929	1958	1995	George Washington
Hallinan, Maureen	1941	1972	1996	Notre Dame
Smelser, Neil	1930	1958	1997	Berkeley
Quadagno, Jill	1942	1976	1998	Florida State
Portes, Alejandro	1944	1970	1999	Princeton
Feagin, Joe	1938	1966	2000	Florida
Massey, Douglas	1952	1978	2001	U of Pennsylvania
Reskin, Barbara	1940	1973	2002	Harvard
Bielby, William	1947	1976	2003	California S. Barbara
Burawoy, Michael	1947	1976	2004	Berkeley
Duster, Troy	1936	1962	2005	Berkeley
Epstein, Cynthia Fuchs	1933	1968	2006	CUNY
Piven, Frances Fox	1932	1962	2007	CUNY
Kalleberg, Arne	1949	1975	2008	North Carolina
Collins, Patricia Hill	1948	1984	2009	Maryland
Glenn, Evelyn Nakano	1940	1971	2010	Berkeley
Collins, Randall	1941	1969	2011	U. of Pennsylvania
Wright, Erik Olin	1947	1976	2012	Wisconsin
Ridgeway, Cecilia	1947	1972	2013	Stanford
Lareau, Annette	1954	1984	2014	U. of Pennsylvania

^a^Names in this table are listed in order of date of presidency; in later tables they appear in other orders, to make it easier to see the patterns on other variables.

^b^These are sometimes other than the ones in which the holders had spent most of their careers.

These people have come to the presidency from a variety of universities, though most of those have some claim to provide leading research departments. They bring to the role long academic records, in which there are many potentially relevant factors, some treated below. The presidency is a late-career position, sometimes reached after formal retirement; only one of the twenty got there before their fifties, and nine of them were 65 or older.

Systematic data on their family backgrounds are not available, but some information is provided by various biographical and autobiographical sources. We note some classic American-dream ascents: the two African-American presidents fought their way up against racial barriers, Etzioni was a German-Jewish refugee who dropped out of high school in Israel to join Palmach, the Jewish elite commando force, before higher education, Glenn spent her early childhood as one of the citizens of US-Japanese origin interned in camps in the 1940s, Reskin was from a working-class family and her father died young, Burawoy’s parents were refugees from Russia then Germany, and his father too died young. They all reached graduate school eventually, but several received their doctorates relatively late for reasons related to such contingencies. Several of the presidents report being found promising academically before the graduate-school stage, and picked out for sponsorship; others started on other tracks, and then desire to change led them back to academia. Those trajectories suggest a somewhat open rather than a closed elite at the early-career stage.

A majority of the presidents had spent some of their higher education in fields formally other than Sociology [Table 2] – in some cases chosen as instrumental to the planned direction of their sociology, in others representing a change from earlier plans by moving into sociology.

**Table 2:** Recipients of earlier disciplinary training in other fields

Smelser	Social Relations [Harvard], PPE^a^ [Oxford]
Piven	City Planning
Duster	Journalism
Feagin	History/Philosophy, Social Ethics
Epstein	Political Science, Law
Randall Collins	Psychology
Glenn	Social Psychology
Ridgeway	Social Psychology
Hallinan	Mathematics, Education
Wright	Social Studies, History [Oxford]
Burawoy	Mathematics, Anthropology
Bielby	Electrical Engineering, Economics
Massey	Anthropology, Psychology, Spanish
Patricia H. Collins	Social Science, Education

^a^Politics, Philosophy and Economics, a longstanding and prestigious Oxford degree program.

Piven’s apparent postdoctoral disciplinary identity has fluctuated over time. In 1966–72 she came under Social Work at Columbia; then from 1972 to 1982 she was in Political Science at Boston University, and her 1973 Guggenheim fellowship is also listed under that head. Of course what she taught can still be regarded as sociology, whatever the title of the department. Several of the presidents have had later formal affiliations with ethnically defined departments: Patricia Collins, African American Studies; Alejandro Portes, Latin American Studies; Evelyn Nakano Glenn, Asian American Studies. It is not clear whether that should be seen more as sidelining them as sociologically marginal, or as placing them where the action is with their own shop to run.

Did these complicated trajectories make the presidents less representative of the discipline? Social Psychology has often been treated as a special subfield within sociology, versions of Mathematics and Social Anthropology have commonly been options or requirements, and so on – and the common complaints against fragmentation of the discipline^12^ suggest that such diversity of paths may have been quite normal, even if felt to be less desirable for disciplinary identity and coherence. We can see that the presidents as a group had very mixed intellectual and personal backgrounds, not all ones helpful in developing a successful career. How [a]typical these backgrounds are cannot be known without comparable information on non-presidents. Clearly, however, few of the presidents have had a straight-down-the-line commitment to ‘sociology’ all the way.

### What Qualifications have the Presidents had for the Presidency?

Despite the broad similarities shown, there are also important differences among the presidents in some features of their intellectual work. It is very noticeable, reading their cvs, that almost all of them come to focus on a particular substantive topic area, often one that relates to their personal origins. The members of ethnic minorities work on those, and immigrants sometimes publish in their languages of origin; Kalleberg, of Norwegian origin, works on Norwegian topics with Norwegian collaborators (and receives a Norwegian honor), Portes from Cuba works on Latino immigrants and often publishes in Spanish - while women work on women (and win Jessie Bernard^13^awards). Women who became president have, along with many others, been active in the feminist movement, which has remained organized and active in ASA politics. Others seem to have fallen into a field more accidentally in relation to personal identity but draw on that field for examples when writing on more general issues. By mid-career there is very noticeable differentiation by specialism, though issues of class, race and gender inequalities in education and work have often been a focus. (To define the issue as inequality is a way in which comparable features can be seen in different substantive areas, and such group differences be felt to call for political action.).

Their cvs show, without needing any formal analysis, that in addition to publishing their own work these people have been active participants in the wider functions of the discipline; they have, for instance, been members of many editorial boards, served on ASA committees,^14^ and been prominent in other learned societies. At least eleven^15^ of the twenty presidents have been elected by their peers to leadership in one of ASA’s sections.^16^ Some have also played advisory roles in government and produced reports which drew on their research, been active in consultancy or promotion of greater equality of access to education or jobs, advised the White House, or acted as expert witnesses. They have also given important named lectures and held visiting professorships, and they have received many awards, both for their intellectual work and for other contributions. Some from the ASA as a whole are listed in Table 3; the awards recognize diverse types of contribution and career trajectory.^17^

**Table 3:** ASA awards received by presidents

Collins, R	Dist	
Portes	Dist	DuBois
Massey	Dist	Pub
Duster	DuBois	
Reskin	DuBois	
Collins, P.	Jess	
Epstein	Jess	
Glenn	Jess	
Ridgway	Jess	
Piven	Prac	Pub
Burawoy	Teach	

*Teach* Distinguished Contributions to Teaching Award, *Jess* Jessie Bernard award, *Dist* Distinguished Scholarly Publication, *DuBois* DuBois-Johnson-Frazier Award, *Prac* Distinguished Career Award for the Practice of Sociology, *Pub* Award for Public Understanding of Sociology.

A measure of the intellectual recognition that their work has received from sources outside ASA and their own universities is given by the special fellowships they have held. Some of the commoner of these are from the Center for Advanced Study in the Behavioral Sciences (CABS), the Guggenheim Foundation (Gugg), and the elite Sociological Research Association (SRA),^18^ all listed in Table 4.

**Table 4:** Selected fellowships held by presidents

Bielby	CABS		SRA
Collins, R.			SRA
Duster		Gugg	
Epstein	CABS	Gugg	SRA
Etzioni	CABS	Gugg	SRA
Feagin			SRA
Hallinan	CABS		SRA
Kalleberg	CABS	Gugg	
Massey		Gugg	SRA
Piven		Gugg	
Portes	CABS		SRA
Quadagno	CABS	Gugg	
Reskin	CABS		SRA
Ridgeway	CABS	Gugg	SRA
Smelser^a^	CABS	Gugg	

^a^There is almost certainly more to mention here, but the cv to which I have access unfortunately does not have an ‘Honors and awards’ section.

(More, some of them important foreign or natural-science distinctions, are also found in the presidents’ cvs, but were too diverse to summarize, so what is listed here does not do them full justice.)

There can be no doubt, thus, that these presidents have been by customary standards distinguished, and have received formal recognition of that. But there are quite a few others who have been active in the same spheres without becoming president. We do not, however, have data on non-presidents sufficient to allow discussion of whether the presidents are by objective standards superior to other distinguished colleagues, or different from them in some other way. In any case it could be that once a certain threshold has been crossed the final stage may be random, socially accidental – or this could be where the cartel (if there is one) triumphs.^19^

However, citations may be used as a measure of intellectual leadership, despite the well-known methodological problems they raise. To be cited is at least to be noticed to some extent, which many articles are not, so citations provide one means of assessing the degree of interest in an author’s work, though it is not clear what the citation facts imply beyond at least partially shared research interests. A crude source of easily accessible summary citation data is the number of citations given in Google Scholar, and here this is used by combining the figures for the five most cited items for each president to make a total score. This gave an average score of 6210, with the lowest at 1361 and the highest at 24,587. Although there is a large difference between the lowest and highest citation totals, it must be assumed that even the lowest figures are very high by population standards, though the *non-*president in the Index sample with the most publications had a citation total of 8202. Is it surprising that among the presidents 14 had their greatest number of citations for a book, leaving only six with their most citations for an article? That does not support Turner’s account of the situation as journal-based.^20^ It is, however, entirely consistent with the more substantial study by Clemens et al. (1995) of patterns of publication and citation.

Since citations cumulate over time, work that has been out longer accumulates more. It is not practicable to treat publications individually, but in a rough and ready equivalent we can relate total citations to date of doctorate to see the effect of this. A glance is enough to see that if there is any such effect it must be competing with other factors; Portes has far more citations than others with doctoral dates within two years of his [Epstein, R. Collins, Glenn, Hallinan, Ridgeway], and authors with the three most recent doctorates [Massey, Lareau, P. H. Collins] have exceptionally high levels of citation rather than the hypothesized lower ones.

There is a marked tendency for both the presidents’ empirical and theoretical work to be relevant to socio-political issues, whether or not the authors make explicitly normative statements. A considerable number of these people were in graduate school during the long sixties, and a glance at their careers shows how several have been active on the political left (Sica and Turner 2005). Bielby lists on his cv major involvement in provision of data used in class actions against Walmart for employment discrimination against women; Etzioni pursuing communitarianism, and Piven working on poverty and welfare, have shown a high level of political commitment in action outside as well as within the academy, and for other candidates besides them it is clear that a left/liberal stance has been favored by the electorate.

All the presidents studied have significant numbers of publications, but these differ markedly in their character, sometimes in surprising ways.^21^ It might be expected that their publications were clustered in the most prestigious journals, especially the ASR, but the data show otherwise; see below.

### Journals and the Presidency

We turn now to look at the journal system and how it is related to employment and ASA politics. It is striking that, as Table 5 shows, six of the presidents (P. Collins, Duster, Glenn, Lareau, Piven, Smelser) had in their whole careers had only one paper in any of the three top journals – and those must be their (unrefereed) presidential addresses. This raises a wider issue about total patterns of publication, as well as numbers. It is interesting to note the patterns among the presidents in the timing of their papers in the top journals – see Table 6 for examples. In addition to the six, Etzioni and Epstein’s one or two top papers decades before the presidency look rather perfunctory, while some of the others have had a fairly steady flow of top papers over their whole careers.^22^

**Table 5:** Numbers of papers by the presidents in top journals before their presidency

Number of top articles published	0	1–5	6–10	11–15	16+
Number of presidential authors	6	2	4	5	3

**Table 6:**
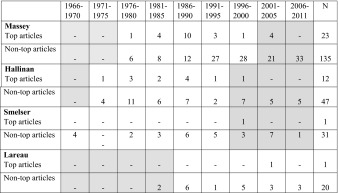
Would they get a job now? Presidential publication trajectories

Shaded cells on the left in this table are for before that president had their PhD, and those on the right are for periods after the presidency

The pattern suggested by Turner’s (2014: 93–5) data from the Sociology Job Market Forum is one where postdocs seeking good jobs would strive to get maximum possible top journal publications, and success without some would be impossible; the six presidents with no top articles would presumably not have got jobs more recently.^23^ No confident statements can be made about the standards represented in the non-top journals used; we can, however, say with confidence that as a set they have less prestige to confer than the top ones. But J. A. Jacobs (2015: 9) found that in 2010–2014 the most *cited* articles were not those in the (generalist) ASR, AJS, or *Annual Review of Sociology*, but in the leading specialist journals. Four cases – Massey, Hallinan, Smelser and Lareau, chosen to exemplify different article publication patterns - are examined more closely.

Massey [president 2001] had by 2001 a truly impressive total of articles, 19 in top journals and 81 in others; many of those have also been reprinted or translated (especially into Spanish). By no means all the non-top journals in which he had published are specifically sociological. Nine or more of his papers have appeared in each of four non-top journals, *Demography*, *International Migration Review*, *Social Science Quarterly* and *Social Science Research*. (He has been a member of the editorial board of all those, so is particularly associated with them.) Of the latter two, with general titles, SSQ on its web site declares its mission as ‘an interdisciplinary journal that publishes high quality, empirical social science research that is of interest to a broad audience of readers across several social science disciplines or which has broad appeal within one discipline. Manuscripts with social and public policy implications and those with comparative or international focus are especially welcome.’ SSR on its web site says that it ‘publishes papers devoted to quantitative social science research and methodology. The journal features articles that illustrate the use of quantitative methods in the empirical solution of substantive problems, and emphasizes those concerned with issues or methods that cut across traditional disciplinary lines’.

The choice of cross-disciplinarity, quantitative method, and social policy relevance, rather than the unequivocally ‘sociological’, is clear. However, his articles have also appeared in a remarkable range of other highly specialized journals, many of which appear only once in his cv. (For details, see Appendix.) It looks possible that the specific topics and journals of his later career may be related to the number and diversity of his collaborators, in a survey- and demography-oriented setting, as well as to his cross-disciplinary^24^ interests.

Sharply contrasted with this are the patterns of Smelser [president 1997] and Lareau [president 2014]. Why, by the end of a long career of the greatest distinction, had Smelser not published more top articles? The answer is surely that he has also registered 17 authored and 21 edited books, plus 54 chapters, in addition to 39 articles in non-top journals!^25^ But that does not really help to answer the question; it seems very unlikely that he submitted more but they were all rejected. One may also note that, as he discusses in an autobiographical paper (Smelser 2000), he deliberately departed considerably from the usual pattern of continuing to develop the same broad area of interest, and has had a consistent preference for interdisciplinary work. For Lareau, it is probably important that her research has rested on intensive and time-consuming ethnographic work, resulting in books that have been very favorably received – one won four different awards - but a relatively small number of separate publications.

Hallinan was a sociologist of education, and by presidential standards moderately prolific in top articles; her career top-article score was twelve. (Although her doctoral work was done at Chicago, only one of those articles appeared in AJS; five were in ASR and six in SF. Is there a story there?) The titles of fourteen of the 23 non-top journals in which she had papers show that they clearly specialize in educational or education-relevant topics, and it is obvious that some are addressing an audience other than that of professional sociologists; in university-department terms, they look more like ‘Education’ than ‘Sociology’. (She did actually receive a PhD in both; in addition her MA was in mathematics, and the few of her papers not on education sound largely technical and mathematical in character.) Almost all her book chapters were also on aspects of education. We may see the levels of specialization that can be reached by comparing the non-top journals in which Hallinan and Lareau have each published at least one article; despite both working on aspects of school education they had only one journal, the *American Educational Research Journal*),^26^ as a common outlet.

These cases are not presented as a typical set, but as exemplifying diverse ways in which the publications system provides opportunities for authors to make decisions on where to publish, adopting different strategies which (in the cases examined) turn out to enable the building of a career reaching the top of the hierarchy. Those decisions will commonly be influenced by the topic areas of their work; Maureen Hallinan was not likely to submit a paper to the *Review of Suicidology*, or David Phillips to the *Journal of Classroom Interaction*, even if each was following the same general policy. But both have had papers in the *Journal of Mathematical Sociology*, a type of journal that provides the opportunity not only to present work directly on a technical methodological problem, but also to abstract from substantive findings to a level of formality independent of them. This can, of course, be done with theoretical approaches as well as with methodological issues; thus Phillips had a paper specifically about ‘Missing features in Durkheim’s theory of suicide’ (Phillips et al. 1993), and Hallinan’s work on classroom interaction drew on wider small group theory. Similarly, both Hallinan and Smelser had papers in *Social Psychology Quarterly*, despite complete absence of any other overlap.

As Table 7 shows, there has been a tendency for those with more top papers also to have produced more other papers, and vice versa, although there is a considerable range of individual patterns. The average number of non-top papers for those with up to five top papers is 31, while for the three with 16 or more top papers it is 103. It does not look, therefore, as if quantity is traded for quality; some people have just been more prolific. It would be interesting to explore whether such patterns influenced status. Perhaps the age at which people became president might serve as an indirect measure? It is indeed the case that those with the highest numbers of top papers became president with an average age of 54, as compared with 66 for those with the lowest numbers; similarly the average for the highest was 28 rather than 35 years since their doctorates.

**Table 7:** Numbers of top and non-top papers by presidents

Non-top papers → Top papers ↓	10–19	20–29	30–49	50–79	80–99	100+	Average
1–5	2	2	2	2	−	−	31
6–15	−	1	33	3	1	1	63
16+	−	−	−	1	−	2	103

Among the presidents, the ratio of non-top articles to ones in the top journals is never less than 2:1; extreme cases are Lareau with 20 to one, and Etzioni with 166 to three. Clearly, then, any description of the publication patterns of this elite group must give attention to non-top journals, and those may be specialized enough to be well known only to others in the intellectual community with which the author’s research is affiliated. A strong numerical bias to publication in journals which focus on areas of specialization does not look like evidence for a single discipline-wide cartel. We may add, since a high number of each president’s publications appeared before they gained presidential office, that they do not seem to have been handicapped by their condescension towards lower-ranked journals.

### How Representative are the Top Journals?

The Index sample throws some light on what has been done by a more representative group of authors than the presidents. There are 126 papers in the Index sample, and 76 different authors responsible for them. The authors of five or more top articles, the ‘Prolifics’ in this context [Grandjean 5, McRae 5, Treiman 6, Hallinan 9, Phillips 12], had produced more than a quarter of the total, and a major difference to the distribution is made by the two top scorers.^27^ A majority of the authors had contributed only one top paper (and no author had three or four). If we look at the distribution between the top (and more general) journals and the more specialized non-top ones in our sample, a pattern of interest emerges: the ‘Prolifics’ have 76 %, a clear majority of their papers, in the top journals, while the others are fairly evenly divided between the two categories. These figures suggest the evolution of a pattern of stratification, whether between individuals or between institutional settings – but there could be other reasons connected with, for example, sub-disciplinary identifications or historical career opportunities.

Specialist fields are relevant here. Doctoral dates could not be found for 30 % of the cases in the Index sample. It is suspected that at least a few of those may have been on a career path based in practice-oriented settings such as Nursing or Education which did not require doctorates,^28^ and available information on several mentioned MDs rather than PhDs; others may still have been students. If so, the missing 30 % are not ‘missing’ for our concern, but make another constituency of interest for the study of journal processes. Google elicited some job titles for sample authors (e.g. Program Specialist, Health and Human Services Commission; Director of Public Education, County Health Department)^29^ to whose holders the possible relevance of sociology was evident, though it seems unlikely that the writing of articles in sociological journals was part of the job description and so likely to continue. Transient membership of the article-writing class, with practitioner issues providing areas of specialization when roles change, could be quite common.

Authors decide where to submit, but editors and the referees they have chosen decide which submissions succeed. If there is a cartel, this could be a key point of connection. Our presidents have held some ASA journal editorships: Neil Smelser, ASR 1963–5; Randall Collins, *Sociological Theory*, 1980–4; Maureen Hallinan, *Sociology of Education* 1982–6; Cecilia Ridgeway, *Social Psychology Quarterly* 2001–2003. We may note that the dates when the ASA editorships were held leave long gaps for the presidents, and more particularly it is by 2016 forty-five years since one of them scaled the summit of power in the ASR.

One can be confident that the presidents, like their contemporaries, have done a fair amount of refereeing over their careers, though no figures on that are available. Editorial board memberships provide the best available substitute data, though boards seem to be used in different ways by different editors – sometimes just to confer status. Departmental journals, which include AJS and SF, have commonly had boards consisting largely of department members, while associational ones like ASR have aimed for diversity and wide representation. Since 1975, for almost all the time at least one of our eventual presidents has been a member of the ASR board, starting with Glenn in 1975, and going on to Portes, Burawoy, Kalleberg, Quadagno, R. Collins, and Quadagno again.^30^ Turner’s treatment of board memberships as political placements on behalf of the cartel ignores editors’ needs to have in their labor force a range of current specialisms covered. Editors need to be prepared for new contingencies, and that does not imply bias. The variety and temporal scatter of those members do not suggest cliquish disciplinary dominance, though they leave some space for cartel action if there is such.

How much of a mark have the presidents made on the total number of papers? If we look at the 1986–1990 period, when all 20 presidents were active and the highest total number of presidential top papers was published (Massey had 10!) – the total was 35; as proportion of the estimated total of c. 620 that does not seem high enough to see it as an expression of cartel dominance. Perhaps it suggests, if anything, an intellectual clique or pressure group rather than a more substantial level of control?

All these points focus on factors influencing the individual authors’ rates and styles of publication. But, as other writers have pointed out, there are also constraints and opportunities at the system level. How many journal articles in total can be published? The number depends on how many journals exist, how often their issues appear, and how many pages they make available for articles of what length. (Turner suggests that a high refusal rate is maintained to support journals’ status ratings.) The market may adjust more or less automatically to changing demand, but it is equally possible that, for instance, growth in numbers of faculty posts increases the felt need of their potential holders for publications, but this makes access to publication more competitive rather than leading to an increase in the number of pages which maintains long-term consistency of opportunities. There is, of course, also a commercial market of journal prices and sales involved.

### Cartel Formation?

Turner’s argument suggests that cartel identities, associated with publication practices, might be the missing factor which distinguishes those elected. He saw these identities and friendships as established in graduate school, then carried forward through whole careers. To check up on that we cannot plot networks of friendship, but we can adduce some other potentially relevant data. If such relationships are formed in graduate school, the presidents’ opportunities to have met there are relevant. Table 8 shows whether they were in the same school around the same time; it is assumed that the five years before graduation is a reasonable estimate of the period when people were there and such relationships could be established. By that criterion, the overlap in graduate school was negligible: Kalleberg and Bielby were at Wisconsin in the same period (and published a joint paper in 1981), − and that’s it. There could also be potential contacts across the student/faculty border; Smelser reached Berkeley in time to overlap slightly with Etzioni, but that is the only such opportunity recorded.

**Table 8:** Were they in graduate school together?

	PhD date	Minus 5	Doctoral university^a^
Etzioni	1958	1956^b^	Berkeley
Collins, R.	1969	1964	Berkeley
Wright	1976	1971	Berkeley
Lareau	1984	1979	Berkeley
Collins, P. H.	1984	1979	Brandeis
Piven	1962	1957	Chicago
Hallinan	1972	1967	Chicago
Burawoy	1976	1971	Chicago
Epstein	1968	1963	Columbia
Ridgeway	1972	1967	Cornell
Smelser	1958	1953	Harvard
Feagin	1966	1961	Harvard
Glenn	1971	1966	Harvard
Quadagno	1976	1971	Kansas
Duster	1962	1957	Northwestern
Massey	1978	1973	Princeton
Reskin	1973	1968	Washington
Portes	1970	1965	Wisconsin
Kalleberg	1975	1970	Wisconsin
Bielby	1976	1971	Wisconsin

^a^They were in departments of sociology unless another disciplinary affiliation is mentioned.

^b^This figure does not mark the 5-year time span – he finished his PhD in the record time of 18 months.

We may note that most of the departments through which this cohort passed in graduate school were undoubtedly elite ones, which could encourage a diffuse sense of shared elite identity. However, these are some of the largest graduate schools, so they would also have been turning out far more future non-presidents, and the demographic squeeze would have made it impossible for many of their students to find posts in equally prominent departments.

If we extend the focus to early membership of faculty in the same department, there were two situations where people were in the same department at the same time. Table 9 shows what their opportunities for getting together then were.

- Kalleberg and Reskin appear together at Indiana.
- Hallinan and Wright overlapped at Wisconsin-Madison (though Hallinan in that period also had two visiting posts elsewhere in Education), and nothing appears to have stemmed from this - which, given their different intellectual styles and empirical topic, is not surprising. Burawoy was also there for one year, and in connection with this short move he established ‘a lifelong friendship and joint commitment to Marxism’ with Wright. (Burawoy 2005: 60)

**Table 9:** Were they in the same departments in their early careers?

	First assistant professorship	Date and next dept
Smelser	Berkeley	[1957- stayed]^a^
Glenn	Boston U	1972–84 [Florida State]
Feagin	California Riverside	1966–70 [Texas]
Duster	California Riverside	1963–5 [Stockholm]
Collins, R	California San Diego	1969 [stayed]
Bielby	California Santa Barbara	1977-[stayed]
Etzioni	Columbia	1958- [stayed]
Piven	Columbia [Social Work]	1966 [stayed]
Portes	Illinois Urbana Champaign	1970–71 [Texas]
Kalleberg	Indiana U	1975–80 [stayed]
Reskin	Indiana U	1973 [stayed]
Quadagno	Kansas	1977–81 [stayed]
Massey	Pennsylvania	1980–85 [stayed]
Epstein	Queens, NY	1968–70 [stayed]
Lareau	Southern Illinois	1986–90 [Temple]
Collins, P H	Cincinnati [African American Studies]	1982–87 [stayed]
Hallinan	Wisconsin-Madison	1972–6 [stayed]
Wright	Wisconsin-Madison	1976–80 [stayed]
Ridgeway	Wisconsin-Milwaukee	1972–8 [stayed]
Burawoy	Berkeley	1976–82 [Wisconsin; Berkeley]

^a^‘stayed’ means that they remained in the same department after the assistant professor stage; when another department is named in the third column that is where they went after the first professorship. Some starting dates in this table are correct, but a bit misleading about potential contacts, since people could hold a variety of temporary posts around campus before getting a professorship.

Once again this does not look like much of a dominant network – or at least not a single one.

Another possible locus or basis of subgroup formation is ASA section membership (Table 10).^31^ In 1990, the presidents belonged to 22 different sections, some to more than one, with five the highest number of presidential members in one section (Marxist Sociology); by 2003 they belonged to 27, with the highest number six (Economic Sociology). This looks as much or more like a considerably divided group than a united one promoting shared interests. The sizes of the sections’ whole memberships suggest that they, especially those most popular with the presidents, were not just small solidaristic groups, and the scatter of affiliations across the range of possibilities, with presidential numbers highest at the summit of total size of section, suggests an element of representativeness of the broader ASA constituency. However, the absence of some very popular sections (Medical, Family, Ageing) from Table 10 indicates areas of non-representation of the constituency. Those sections are on topics perhaps more likely to interest colleagues with an orientation to practitioner roles than to macro problems and ones internal to the discipline.

**Table 10:** ASA section membership, 1997–8 (Rosich 2005:145)

Section	Sample members	Total membership, 1995^b^
Race, Gender and Class	Reskin	[no figure given]
Rational choice	Collins R.	205
Law	Epstein	305
Emotions	Collins R.,* *Ridgeway*	308
International Migration	Portes**	326
Asia, Asian-American	Glenn*	330
Children	Lareau*	378
Marxist	Burawoy, Feagin, Wright	406
Science, Knowledge	Duster	407
Methodology	Bielby	410
Political Economy of the World System	*Portes*	410
Education	Collins R., Hallinan,* Lareau*	519
Community and Urban	Massey, Portes**	538
Comparative, Historical	Glenn, Quadagno	538
Collective Behavior, Social Movements	Smelser	549
Political	Quadagno*, Wright	554
Aging	Quadagno	560
Social Psychology	Ridgeway**	619
Theory	*Collins R.* *, Epstein, Ridgeway, Smelser	749
Racial and Ethnic Minorities	Feagin*	865
Culture	Bielby	865
Organizations & Occupations	Bielby, *Epstein*, Kalleberg, *Reskin*	936
Peace, War and Social Conflict	Collins R.*	
Sex and Gender	Epstein*, Glenn, Reskin*, Ridgeway	1317

*Italicised names* identify those known to have acted as chair or president of that section*;* asterisks indicate that they won a distinguished book or career award from that section^a^

^a^The asterisks which appear here considerably under-value the number of such awards, because some relevant sections no longer exist, and awards have sometimes been given to non-members of the awarding section.

^b^Rosich (2005):145).

A more direct indicator of networks than shared section membership could be seen in various forms of collaborative work, some of which appear in cvs. The most actively collaborative items found are joint publications:

- Kalleberg and Reskin, who overlapped at Indiana, had two joint authorships. (1995, 2000); Reskin has contributed a chapter to a book edited by Kalleberg (2001), and they have contributed separate chapters to the same two (1997) edited volumes.
- Burawoy and Wright have two joint publications (1990, 2003)
- Bielby included gender in his research on work as the result of an approach from Reskin (Friedland 2002).
- Epstein and Kalleberg have had two joint editorships (2001, 2004) without shared departmental membership.

Less actively collaborative are parts of the compilation of collective works:

- Ridgeway, Portes and Reskin have all contributed to an encyclopaedia (2001) edited by Smelser [but so have a large number of other people]
- Wright has contributed to a 2008 book jointly edited by Lareau
- Massey has a paper in a (2000) volume jointly edited by Smelser
- R. Collins has contributed to a 2000 handbook edited by Hallinan
- P. Collins has a chapter in a 1994 book edited by Glenn
- Glenn has a chapter in a 2007 book jointly edited by Burawoy.

Paula England, 2014 ASA president and a recent ASR editor, demands a mention here as a possible missing link: she has jointly (with R. Collins and others) edited a collection (Guillen et al. 2002) to which Portes, Reskin, Ridgeway and Lareau all contributed; she also had a joint article (2007) with Ridgeway.

This looks more like a network, but it does not look like one to which every president belongs; names which do not occur at all include Duster, Etzioni, Feagin and Piven, while others suggest a rather slight link. Those involved could be long-standing and close friends with a shared political agenda - but they could also have never met in person, or have been invited in order to contribute a different perspective from the editor’s; they could be a group clearly distinguished from potential rivals - or could be just a few among a larger number of non-presidents who have also been invited to contribute to the same edited collections. There is nothing unusual in people who have worked in related areas citing each other’s work, or their papers being jointly authored. It is not clear what these factual links can be taken to imply beyond the existence of overlapping research or teaching interests.

A really effective cartel would, consciously or not, take successful steps to perpetuate its dominance. What might this imply in relation to the ASA? Clearly the electoral system gives the decisions of the Committee on Nominations great importance, and one might expect attempts to be made to ensure a reliable membership of it. However, for the period 2004–2013, for which election results are given on the ASA web site, not a single member of our group of presidents became a member of it; this could indicate either surprising unpopularity with the electorate, or careless disregard of this key niche in the political system. However, less senior cartel members, who we cannot identify, may have been there.

Another place where cartel activity in relation to journals might be expected is the membership of editorial boards. It is easy to collate information on their membership, though the results may be of limited use as data, since boards are used in different ways by different editors – sometimes as a source of general policy and responsibility for heavy refereeing loads, sometimes just as decoration and claims to status.^32^ Department-based journals have tended to have editorial boards dominated by department faculty members, though in recent years some have become more open and/or have added international representation. Here we look simply at the memberships of the top-journal boards held, at five-yearly intervals, by the 20 presidents.^33^ First we see that nine of the presidents show no such memberships. Then we note the departmental role: Kalleberg served SF from1990 to 2005 – but he was a member of its department then, as was Massey for AJS in 1985 and 1990 when at Chicago. Randall Collins had the highest other score, working for ASR in 1995 and 2005 and for SF in 1980. How does this compare with non-presidents? There were 97 who had belonged to more than one of the boards, seven of whom had belonged to three. The only individuals who had served for all three top journals were Ronald Breiger, Paul Burstein, Jack P. Gibbs, Darren Sherkat and Mayer Zald [all at Vanderbilt], who together filled 16 slots. We do not know the mechanisms by which those editorial board members were recruited, but none of them have been presidents.

## Conclusions

Some general characteristics of the presidents and their publication careers have been reviewed, and we can see that those who became ASA president in the last 20 years had been in a variety of ways intellectually prominent among US sociologists but, despite that, had also been very diverse in their backgrounds and career styles. How far have they fitted the model of either an elitist cartel, or a group that can be used to characterize US sociology as a whole?

The data on graduate-school timing make it seem very unlikely that a long-term cartel affiliation was established in graduate school for those who later became president, even if it may have been for wider groups. The social pattern of joint publications sketched can quite plausibly be seen as one manifestation of a meaningful, if not very strong, network, but if it is there has been at least a minority, maybe a majority, of the presidents who do not appear to have belonged to it. Does the presidents’ range of interests correspond to that of ASA members or the discipline as a whole? Yes to some extent. (To expect anything like the full range of ASA members’ interests to be represented by any list to which only one new person is added each year, and older members are at late career stages or fully retired, would not seem realistic.) As Turner (2014: 109–112) points out, there appear to be shared political norms which encourage a preoccupation with inequalities, and that preoccupation is salient across substantive topics which otherwise appear as different; thus the presidents’ work on inequalities in schools and in the occupational sphere is indeed somewhat representative. (cf. Jacobs 2004).

Our finding about the presidents’ higher citation scores for books than articles may not destroy the perception of top-journal articles as being especially important, but it surely weakens it a little. What weakens it more is the high presence of non-top journals, and in some cases the near absence of top journals, among those in which the presidents have published; any description of the publications system and the presidency which does not give serious attention to that sector is surely missing out a significant part of the picture. That seems associated with the emphasis, among most if not all of the presidents, on areas of research specialization and their associated journals. As Turner himself points out, there are also large numbers of sociologists at non-elite departments who have continued researching and publishing in areas outside the ASR nexus. The persistent research focus on only two or three generalist journals is convenient to the historical researcher, but has left unanswered questions about whether there are meaningful differences in content and topics, methodological adequacy, theoretical orientation, audience addressed, between these journals and others. Work on the history and sociology of sociology will be more informative if it takes into account a larger part of the work actually done.

## Appendix

Index

The intention of the compilation of the ASA’s *Cumulative Index of Sociology Journals 1971–1985* (Lantz 1987) was bibliographical, but its material can be treated as in some sense a sample of authors, of articles and of journals. The exercise started as the indexing only of ASA journals, but then added AJS and SF, without the resources to go further. The journals eventually covered are thus AJS, ASR, *The American Sociologist*, *Journal of Health and Social Behavior*, *Social Forces*, *Sociometry/Social Psychology Quarterly,* 34 *Sociological Methodology*, *Sociological Theory*, *Sociology of Education* and *Contemporary Sociology*. 1971 was probably chosen as the starting point just because that was when the proposal to create the Index was accepted; the originally planned ten-year span then grew to 1985, which was as near as practicable to the then-present day. That 15 years may have some historical specificity, which makes it desirable to be careful about generalizing from it to other periods.

The content of the Index is a list, alphabetical by surname, of every author of a publication in the journals covered, with a complete list of their publications in them. It does not provide data on authors’ ages, doctoral dates, ASA membership, training, co-authors, or affiliations when the article was published; where identified^35^ those have been added to what is given in the published article. For the purposes of this paper, the journals generally recognized as the leading [‘top’] ones – ASR, AJS, and SF – are those mainly used.

Only the articles [including research notes] are sampled here. Comments and replies of up to 4 pages were excluded unless they presented new data, and such material as review articles or encyclopedia entries was assumed not to be presenting new findings or ideas and so was also excluded. Revised editions of books, or translations, were not counted.

The sample drawn was all the *articles* listed for the author first named on each right-hand column of the two-column pages; if that author had no articles they did not join the sample, even if they had, for instance, a number of book reviews listed. The finding does not imply that others, listed or not, were failing to participate in the advancement of the discipline; many of them are likely to have contributed to *other* journals [see below], or to have written books, published critical comments etc. The journals whose content is listed are important ones, but there are many others, particularly in specialized fields, in which authors who may look weak here have published copiously; equally there are of course books, presenting both textbook material and new empirical and theoretical work.

**Table:**

Other journals to which presidents have contributed.	
Douglas Massey.	
Journals other than AJS/ASR/SF in which Massey had by 2010 at least one article, with the number of his articles in each.	
*American Law and Economics Review*	1
*American Philosophical Society Proceedings*	1
*Annals of the American Academy of Political and Social Science*	4
*Artes de Mexico*	1
*City and Community*	1
*Demography*	13
*Estudios Demograficos y Urbanos*	1
*Ethnic and Racial Studies*	2
*EurAmerica*	1
*European Sociological Review*	1
*Hispanic Journal of Behavioral Sciences*	1
*International Journal of Conflict and Violence*	1
*International Journal of Group Tensions*	1
*International Migration*	4
*International Migration Review*	13
*Journal of American History*	1
*Journal of Catholic Social Thought*	1
*Journal of Latin American Studies*	1
*Journal of Population Economics*	1
*Kölner Zeitschrift…*	1
*Latino Review of Books*	1
*Latino Studies*	1
*Mexican Studies*	1
*Migracion y Desarrollo*	1
*Migration Today*	1
*Papeles de Poblacio*	1
*Perspectives*	1
*Population and Development Review*	3
*Population Index*	3
*Population Research and Policy Review*	3
*Population Studies*	1
*Proceedings of the National Academy of Sciences*	1
*Qualitative Sociology*	1
*Race and Social Problems*	1
*Revista de Ciencias y Humanidades de la fundacion…*	1
*Revista Espanola de Estudios Sociologicos*	1
*Revista Internacional de Sociologia*	1
*Science*	1
*Social Problems*	3
*Social Science Quarterly*	10
*Social Science Research*	9
*Social Service Review*	1
*Sociological Inquiry*	1
*Sociological Methods and Research*	1
*Sociology and Social Research*	3
*Soziale Welt*	1
*Teaching Sociology*	1
*The American Sociologist*	1
*The Dubois Review*	2
*The Next American City*	1
*U. of Pennsylvania Law Review*	1
*Urban Affairs Quarterly*	2
*Urban Affairs Review*	2
*Urbana*	1

Neil Smelser.

Journals other than AJS/ASR/SF in which Smelser had by 2010 at least one article, with the number of his articles in each.

**Table:**

*American Behavioral Scientist*	1
*American Behavioral Scientist*	1
*British Journal of Sociology*	1
*Cahiers de recherche sociologiques*	1
*California Journal of Politics and Policy*	1
*Contemporary Sociology*	1
*Current Sociology*	1
*Development and Change*	1
*Economic Development and Cultural Change*	1
*Explorations in Entrepreneurial History*	1
*Humboldt Journal of Social Relations*	1
*Informationen*	1
*International Journal of Psychoanalysis*	1
*International Social Science Journal*	2
*International Sociology*	3
*Journal of Classical Sociology*	1
*Journal of the American Medical Assoc*iation	1
*Kölner Zeitschrift*…	1
*La Revue Tocqueville*	1
*La Ricerca Sociale*	1
*Laboratorio di Scienze del Uomo*	2
*Rationality and Society*	1
*Revista Italiana di Scienza Politica*	1
*Social Psychology Quarterly*	1
*Social Research*	1
*Sociologica*	1
*Sociological Inquiry*	1
*Sociological Perspectives*	1
*Sociological Problems*	1
*Sociological Quarterly* [tr. to Russian]	1
*The American Sociologist*	3
*The Journal of Labor History*	1
*The Tieline*	1
*WzB Mitteilungen*	1

Maureen Hallinan.

Journals other than AJS/ASR/SF in which Hallinan had by 2010 at least one article, with the number of her articles in each.

**Table:**

*American Education Research Journal*	1
*American Journal of Education*	1
*Catholic Education*	3
*Psychological Reports*	1
*Child Development*	1
*Curriculum Inquiry*	1
*Educational Administration Quarterly*	2
*Studies in Educational Evaluation*	1
*High School Journal*	1
*Journal of Applied Sociology*	1
*Journal of Classroom Interaction*	1
*Journal of Education for Teaching*	1
*Journal of Mathematical Sociology*	1
*Journal of Research on Adolescence*	1
*Journal of Social Issues*	1
*Ohio State Law Review*	1
*Social Networks*	1
*Social Psychology of Education*	3
*Social Science Research*	1
*Sociological Quarterly*	1

Annette Lareau.

Journals other than AJS/ASR/SF in which Lareau had by 2010 at least one article, with the number of her articles in each.

**Table:**

*American Educational Research Journal*	1
*Childhood*	1
*Education and Urban Society*	2
*Educational Policy*	2
*Elementary School Journal*	1
*Journal of Marriage and Family*	2
*Phi Delta Kappa*	1
*Poetics*	1
*Qualitative Sociology*	1
*Social Problems*	1
*Sociological Forum*	1
*Sociological Theory*	1
*Sociology of Education*	4
*Teaching Sociology*	1
*Theory and Society*	1

## Footnotes

1 Precise data on the numbers are not available, and there have been changes over time in eligibility and in subscription rates which have had their effects, but that there have been enough non-members for treating members as the whole constituency to be misleading is not in doubt (Williams 1982).

2 Two candidates, selected by an elected nominations committee, are offered to the voters, but others can be written in, and sometimes are. (Maureen Hallinan, from the cases used below, was elected as a write-in candidate in 1996.) Categories of membership, and thus the electorate, have, however, varied over time. Until 1982 eligibility for full membership rested mainly on the possession of a relevant doctorate, but this was changed to simply having an interest in sociology (Rosich 2005: 3). Simpson and Simpson (2001: 281–5) show that the proportion of members voting sank from 62.3 % in 1952 to 32.4 % in 1992, while ASA officers less often came from elite departments. The latter is seen by the Simpsons as regrettable for the discipline, part of a movement from scholarly society to professional association - but it does of course make the officers more typical of the membership. Voting rates rose again to 45.3 % (declared comparatively a high rate) in the 2014 election. Whatever the overall rate, it appears that those members of higher status and more integrated into the association’s activities are more likely to use their votes (D’Antonio and Tuch 1991, Ridgeway and Moore 1981); presumably that may reflect ignorance of the candidates as much as disaffection among the less involved.

3 ‘should’ here is used to mean ‘may be assumed to’, not ‘ought to’.

4 See Rosich (2005: 63–65 0 for an account of one major controversy over this.

5 Weeber (2006) summarises past work and provides much data on this, and Baughman (1974) and Turner (2014) have considerable discussion of the situation now. Whatever the specific methods used, the results seem essentially similar.

6 A somewhat analogous argument was used by Yoels (1971), who documented the numerical dominance of graduates from Chicago, Columbia and Harvard in the editorship of ASR. Straus and Radel (1969) addressed a similar issue, arising from complaints at the perceived over-representation of some regions in ASA positions held. (The positions considered ranged from president and vice-president down to Council member elected from affiliated society, and were given weights for their power and importance.) It was concluded, on the basis of painstakingly detailed empirical data, that the South was over-represented and the Midwest under-represented in relation to their productivity and citation eminence; the possibility was raised that this arose from the application of a regional ‘equity principle’.

7 Valuable exceptions to that are Glenn (1971) and D. Jacobs (1989).

8 The only examples of this that I have found are from *Rural Sociology’s* sturdily positivistic papers on its own history: Sewell (1965) and Stokes and Miller (1975).

9 Stinchcombe and Ofshe (1969) had earlier argued that general experience of the lack of reliability of qualitative measurement, such as that used in refereeing and editorial judgment, predicted such findings.

10 See the Appendix for full details of the Index sample.

11 The Index policy of making no distinction between single and multiple authorship is here followed throughout; if Jane Bloggs is sixth author of an article, that is counted as one of her articles. This is inevitably somewhat misleading, because the same article counts six times - but so are the alternatives.

12 See, for instance, a number of papers in Cole 2001.

13 ‘in recognition of scholarly work that has enlarged the horizons of sociology to encompass fully the role of women in society.’

14 Four held other major ASA offices before the presidency: Secretary, Kalleberg, 2002–4; Vice-President, Smelser 1975, Reskin 1991, Quadagno 1993.

15 The uncertainty of ‘at least’ here is because some presidents did not cover such topics in their cvs.

16 For some details see Table 10.

17 Not all of these awards have existed for the whole period of the presidencies covered, so there are some which could not have been held by the earlier presidents.

18 This is an honorific body to which members have to be elected. One president told me that she had not even heard of it until after her election as president.

19 It would be possible to approach this by comparing those elected with defeated candidates for the office, but that has not [yet] been done. It would be harder to collect the data, since presidents appear in more public lists and get special documentation.

20 Jacobs (2007: 128) points out that there were far more relevant books per year than articles in ASR and AJS, so that higher numbers of citations to them are in that sense not surprising. Perhaps it is relevant that a book has more in it, offering more that might be cited? If so, book citations should rise in proportion to length; another potential paper there. But Cronin and Snyder (1997) have already published a paper in which they report finding that there was little overlap between journal and monograph samples in the most cited authors, and suggest that there may be two distinct populations here.

21 Book chapters are clearly important, and written in large numbers by the presidents, but hard to classify meaningfully. (The difficulty of doing so is manifest in the presidents’ cvs, where some put articles and chapters under the same heading, while others introduce categories like ‘short essays’, or asterisk items that have been refereed.) Chapters are commonly seen as distinguished from articles in being invited by book editors, and not subject to refereeing – though some books do use a form of refereeing – and so lacking the certification that articles give. But not all chapters are written in response to invitation, and it is common for them to have appeared initially in a [refereed] journal. It then seems honorific for a book editor to find your article worth reproduction – but the editor may also include some of her own previous articles. In addition, some chapters are deliberately commissioned to review an area of work rather than to make an original contribution. These considerations have led to the omission here of detailed discussion of chapters.

22 The publication pattern of David Phillips, the most prolific of the authors sampled from the Index, shows a particularly extreme trajectory. From 1972 to 1985 he had ten top papers as well as many non-top ones, but after that he had a large number but *only* non-top ones, almost all of which specialised in health and medicine – in journals outside sociology, though some of which we may safely assume were ‘top’ in health and medical circles.

23 Logan (1988) showed that 1130 authors had only one top paper in 1975–1986, leaving 575 papers from those who had more than one.

24 What counts as sociology may be contested, which would redefine what was cross-disciplinary. Librarian Baughman (1974: 302) found that ‘The field is so closely related to other disciplines that one-half of the most heavily cited journals are those which primarily emphasize another discipline’. In sociology submissions to the British Research Assessment Exercise in 2008 the journal articles came from 847 different journals; Piriou and Cibois (2009) found that the articles of a sample of about 300 French sociologists had appeared in 735 journals, few of them the national general ones and many with little obvious connection with sociology.

25 The list of his papers shows a classic late-career pattern, with revised editions, forewords, obituaries and encyclopaedia entries coming to the fore. Many of these do not fit the operational definition of ‘article’ used here when counting totals, so he was more active then than Table 10 suggests.

26 Collyer **(** 2012:228–235) uses contrasts in the pattern of papers cited by people working in the same general field to define broad intellectual cleavages; that approach could be tried in this area.

27 This is analogous to the pattern found by Aksnes and Sivertsen (2004), where whole national scores are affected by the contribution of outlying stars.

28 Or they could, like Patrick Doreian, come from a foreign background where a professor did not require a PhD.

29 Identified via Google’s provision of his mention in a local newspaper as commenting on the potential danger of some lead-glazed dishes on sale.

30 Not all cvs mention such roles, so there could have been more.

31 Some of the membership patterns seem surprising, given what is shown by the pattern of published work – e.g. Massey published extensively on Mexican migration to the US, but did not at that time belong to the Section on International Migration - so it would be unwise to infer that this list gave a full account of people’s long-term interests or socio-intellectual groupings.

32 Abbott (1999) casts some interesting light on internal developments of the Chicago department in dealing with AJS.

33 It should be noted that the younger presidents have had fewer years in which they were eligible to act as referees but, as Table 1 shows, even the two with the latest doctorates were in principle available for 25 years.
